# Phenotypic and genotypic characterization of probiotic strains in the context of antimicrobial resistance

**DOI:** 10.3389/fvets.2025.1684650

**Published:** 2025-10-03

**Authors:** Ádám Kerek, Nikolett Palkovicsné Pézsa, Eszter Kaszab, Ákos Jerzsele, Orsolya Farkas

**Affiliations:** ^1^Department of Pharmacology and Toxicology, University of Veterinary Medicine, Budapest, Hungary; ^2^National Laboratory of Infectious Animal Diseases, Antimicrobial Resistance, Veterinary Public Health and Food Chain Safety, University of Veterinary Medicine Budapest, Budapest, Hungary; ^3^Faculty of Health Sciences, One Health Institute, University of Debrecen, Debrecen, Hungary; ^4^Department of Microbiology and Infectious Diseases, University of Veterinary Medicine, Budapest, Hungary

**Keywords:** probiotics, *Enterococcus*, *Bacillus*, *Lactobacillus*, *Pediococcus*, antimicrobial resistance, ARG, next-generation sequencing

## Abstract

**Introduction:**

Antimicrobial resistance (AMR) has emerged as a critical global public health concern, particularly with regard to microorganisms used as probiotics in both veterinary and human healthcare. The aim of this study was to characterize the phenotypic and genotypic resistance profiles of several industrially applied probiotic bacterial strains, with special emphasis on the presence of antimicrobial resistance genes (ARGs).

**Methods:**

Five strains, *Enterococcus faecium, Bacillus licheniformis, Bacillus subtilis, Lactobacillus rhamnosus*, and *Pediococcus acidilactici* were analyzed. Minimum inhibitory concentration (MIC) testing revealed resistance in multiple strains to clinically relevant antibiotics such as gentamicin, amoxicillin, tylosin, and florfenicol.

**Results:**

Whole-genome sequencing identified 27 distinct ARGs, primarily associated with efflux pumps and target protection or modification mechanisms. The *Bacillus licheniformis* strain harbored the most diverse ARG profile, whereas no resistance genes were detected in *Pediococcus acidilactici*.

**Discussion:**

These findings highlight the necessity of integrating phenotypic and genotypic assessments for the safe application of probiotic strains in animals and potentially human use.

## Introduction

In recent decades, bacterial antimicrobial resistance (AMR) has emerged as one of the most pressing global public health challenges under the One Health framework (1). The excessive or improper use of antimicrobial agents has significantly contributed to the widespread development of resistance (2), leading to a growing interest in antibiotic alternatives. These include antimicrobial peptides (3), propolis (4–6), plant essential oils and extracts (7), as well as medium-chain fatty acids and triglycerides (8), all of which, when applied alongside appropriate biosecurity measures have resulted in a marked reduction in antibiotic usage (9).

The term “probiotic” was first introduced in 1974 to define live microorganisms that, when administered in adequate amounts, confer a health benefit on the host (10). The use of antibiotics as growth promoters, once common practice, was soon associated with the acceleration of AMR development. Consequently, this practice has been banned in many parts of the world, including the European Union, Canada, the United States, China, and Thailand (2). Within food-producing sectors, the highest antibiotic consumption is reported in swine farming (11), followed by the poultry industry (12).

Beyond their industrial relevance, probiotic strains are increasingly recognized for their role in mitigating antimicrobial resistance across diverse settings, including companion animals and human health.

Probiotics have thus been positioned as alternative performance enhancers, aiming to replicate the benefits of antibiotics without contributing to resistance (13). However, evidence regarding their efficacy remains mixed, and to this day, they are marketed mainly as complementary agents rather than primary interventions (14, 15).

In the search for viable alternatives to antibiotics, probiotics offer a multifaceted solution by supporting gut health, preventing pathogen colonization, and potentially reducing the need for therapeutic antimicrobials.

Concerns have been raised that some probiotic strains may harbor antimicrobial resistance genes (ARGs), which may be transferred to the gut microbiota or enteric pathogens via horizontal gene transfer (HGT) (16–18). In the context of the One Health approach, monitoring the ARG content of probiotic strains is critical, as these organisms may serve as reservoirs of resistance, comparable to wild birds in their ecological role (19).

According to the guidelines of the World Health Organization (WHO), the Food and Agriculture Organization (FAO), and the European Food Safety Authority (EFSA), probiotic strains intended for use in food-producing animals must meet defined safety, functional, and technological criteria (11, 20, 21). A key safety concern in probiotic evaluation is their potential to transmit ARGs to the gut microbiome. It is essential that probiotic strains do not act as vectors for resistance genes, particularly those targeting clinically important antimicrobials (22). While stringent regulations exist for food-producing animals, which require antimicrobial susceptibility testing as part of probiotic strain approval (23), no equivalent framework currently governs probiotics intended for companion animals.

It is important to distinguish between intrinsic (non-transmissible) and acquired (transmissible) resistance in probiotic strains. In some cases, antimicrobial resistance may be desirable, allowing probiotic to survive concurrent antibiotic treatment. However, resistance genes may be transferred vertically or horizontally between microorganisms, and the gastrointestinal tract of mammals provides favorable conditions for gene transfer (24).

The beneficial effects of probiotics on host health are mediated through multiple mechanisms. Among the best characterized is competitive exclusion, whereby probiotic microorganisms compete with pathogens for adhesion sites and nutrients in the gastrointestinal tract. Additionally, probiotics may exert direct antimicrobial effects by producing metabolites such as short-chain fatty acids (SCFAs) and can interfere with pathogen metabolism and toxin production. These mechanisms collectively contribute to the modulation of gut microbiota composition in a favorable direction. Moreover, probiotics strengthen the host's intestinal barrier function, enhance mucus secretion, and influence gut-associated lymphoid tissue (GALT), thereby participating in immune modulation (25).

*Lactobacillus*, one of the most prominent probiotic genera, is widely applied across species to support digestive health and improve performance. Several studies on neonatal and weaning piglets have demonstrated the immunomodulatory, antioxidative (26), and growth-promoting effects of *Lactobacillus rhamnosus* strains (27). A meta-analysis of 196 trials confirmed overall positive effects on growth and intestinal morphology (28).

While *Lactobacillus* species generally show susceptibility to penicillins, they exhibit intrinsic resistance to aminoglycosides, glycopeptides, and nucleic acid inhibitors (29). Generally, members of this genus are susceptible to penicillins and β-lactamase inhibitors but show reduced sensitivity to cephalosporins. However, penicillin G resistance genes have been identified in several isolates in various studies (30–32).

The *Bacillus* genus is also of high relevance due to its spore-forming ability, which ensures stability during processing and storage (33). Their supplementation has been linked to improved performance (34), and modulation of gut microbiota in poultry and swine (35). Studies indicate that *Bacillus* species rarely carry ARGs and generally display broad susceptibility to both narrow- and broad-spectrum antimicrobials (36). Some research has highlighted their particular sensitivity to fluoroquinolones, and reduced susceptibility has been observed for older antibiotic classes, which only minimally inhibited their growth (37).

*Pediococcus* strains have also demonstrated noteworthy health-promoting effects. In a study using *Pediococcus acidilactici* FT28 isolated from swine feces, piglets supplemented with this probiotic exhibited significantly improved feed conversion ratios compared to unsupplemented controls. Hematological, biochemical, and antioxidant parameters were also enhanced in the treated group. Recent studies indicate that *Pediococcus* strains generally exhibit resistance to β-lactams, macrolides, and phenicols, and moderate resistance to lincosamides and fluoroquinolones, while often displaying full resistance to tetracyclines (38).

Among companion animal probiotics, *Enterococcus faecium* is one of the most commonly used strains. It is also widely applied in food-producing animals as it possesses numerous beneficial traits, including resistance to gastric acidity and bile salts, and strong mucosal adherence. Supplementation with *Enterococcus faecium* SF68 in weaned piglets has been shown to reduce the incidence of diarrhea and significantly increase villus height and the villus-to-crypt ratio throughout the small intestine compared to control animals in broiler chickens, the *Enterococcus faecium* YQH2 strain was found effective in preventing *Salmonella typhimurium* infection (39). While the pathogen typically reduces body weight and causes morphological damage to the liver and intestine, probiotic-supplemented birds maintained normal growth and showed less pronounced pathological changes (39, 40).

However, several studies have reported antimicrobial resistance in *Enterococcus faecium* strains. Resistance patterns vary depending on the strain, and many are mediated by acquired ARGs (41, 42). In recent decades, vancomycin-resistant *Enterococcus* (VRE) strains have become increasingly prevalent, frequently causing hospital-acquired infections (43). In one study, *Enterococcus* species isolated from cheese were evaluated for tetracycline resistance. Genotypic analysis revealed the presence of *tetM* genes in most isolates and *tetS* in one case (44).

Advances in functional genomics enhance our ability to ensure food safety and product quality, improving the predictability of probiotic health effects. Excluding strains with potentially transmissible ARGs from industrial use minimizes food safety risks (45). In the metagenomic era, increasing efforts are being made to map the genetic makeup of probiotic strains and investigate the expression of their functional properties. This includes examining their adaptation via HGT, evolutionary dynamics, and their potential role in the global antimicrobial resistance crisis. Based on these considerations, our study aimed to characterize phenotypic and genotypic antimicrobial resistance profiles of swine-derived probiotic strains, in alignment with the One Health framework.

## Materials and methods

### Origin of cultures

Five probiotic bacterial strains were kindly provided by the Hungarian Dairy Research Institute. According to the supplier, the strains were originally isolated from the feces of healthy swine. No additional background information is available. The strains included *Enterococcus faecium* (NCIMB10415), *Bacillus licheniformis* (DSM5749), *Bacillus subtilis* (DSM5750), *Lactobacillus rhamnosus* (DSM7133), and *Pediococcus acidilactici* (DSM16243) were swine origin. Species identity of all isolates was confirmed using matrix-assisted laser desorption ionization–time of flight mass spectrometry (MALDI-TOF MS) at the Department of Epidemiology and Microbiology, University of Veterinary Medicine Budapest (Flextra-LAB Ltd., Budapest, Hungary).

### Determination of minimum inhibitory concentration (MIC)

Stock solutions of antimicrobial agents (Merck KGaA, Darmstadt, Germany) were prepared following the guidelines of the Clinical Laboratory Standards Institute (CLSI) (46). The standard stock concentration used for all agents was 1,024 μg/mL, adjusted for purity as specified by the manufacturer, and serial 2-fold dilutions ranging from 1,024 to 0.001 μg/mL were prepared. Sixteen antibiotics were tested, selected based on their relevance to both animal and public health, ensuring a broad representation of antimicrobial classes.

Penicillin, amoxicillin, and amoxicillin-clavulanic acid (2:1 ratio) were dissolved in 0.01 mol/L phosphate buffer at pH 7.2. Ceftriaxone, oxytetracycline, doxycycline, gentamicin, clindamycin, tiamulin, and tylosin were dissolved in distilled water. For the sulfonamide combination (trimethoprim and sulfamethoxazole at a 1:19 ratio), sulfamethoxazole was dissolved in hot water with a few drops of 2.5 mol/L NaOH, and trimethoprim was dissolved in distilled water containing 0.05 mol/L HCl. Gatifloxacin was prepared using distilled water with a few drops of 1 mol/L NaOH. Florfenicol was dissolved using a mixture of 95% ethanol and distilled water, while metronidazole was solubilized with a few drops of dimethyl sulfoxide (DMSO) and distilled water. The term “potentiated sulfonamide” refers to the fixed combination of trimethoprim and sulfamethoxazole in a 1:19 ratio, which was tested as a single antimicrobial agent in this study.

Phenotypic antimicrobial resistance was evaluated by determining the MIC values for each bacterial strain, based on the CLSI standard microdilution method (46). Clinical breakpoints, where available, were interpreted according to the European Food Safety Authority (EFSA) panel on additives and products used in animal feed (FEEDAP) (47). For *Enterococcus faecium*, microbiological cut-off values established by EFSA for probiotic strains were primarily applied. In cases where EFSA did not provide a cut-off (e.g., penicillin), the corresponding CLSI clinical breakpoints were used instead (Supplementary Table 1).

Bacterial strains stored at −80 °C were cultured 24 h prior to testing by inoculation into 3 mL of cation-adjusted Mueller-Hinton broth (CAMHB; Biolab Ltd., Budapest, Hungary) and incubated at 37 °C for 18–24 h. MIC determinations were performed using 96-well microtiter plates (VWR International, LLC., Debrecen, Hungary). Each well of the plate was filled with 90 μL of CAMHB. The first column received a half-dilution of the antibiotic stock solution (initial concentration of 512 μg/mL), followed by 2-fold serial dilutions across the plate, leaving the last two columns as controls. The penultimate column served as a positive control (containing bacteria and medium without antibiotic), while the final column served as a negative control (containing only broth). The quality control strain (*Enterococcus faecalis* ATCC 29212) yielded MIC values within the CLSI-recommended QC ranges.

Bacterial suspensions were adjusted to a 0.5 McFarland standard using a nephelometer (CheBio Developer Ltd., Budapest, Hungary) and added to the wells up to the positive control column (47). After 24 h of incubation at 37 °C, MIC values were read using an automated SWIN MIC reader and VIZION system (CheBio Developer Ltd., Budapest, Hungary).

### Next-generation sequencing and bioinformatic analysis

Genomic DNA was extracted from the probiotic formulations using the Quick-DNA Fecal/Soil Microbe Miniprep Kit (Zymo Research, according to the manufacturer's protocol. Paired-end sequencing was performed using an Illumina NextSeq 500 platform (Illumina Inc., San Diego, CA, USA) (48).

For DNA library preparation (Supplementary Table 2), 7.5 μL of Nextera PCR Master Mix was combined with 2.5 μL each of i5 and i7 index primers and added to the tagmented DNA samples for PCR amplification. The PCR program included an initial denaturation at 95 °C for 30 s, followed by 12 cycles of 95 °C for 10 s, 55 °C for 30 s, and 72 °C for 30 s, with a final elongation at 72 °C for 5 min. Samples were then cooled to 10 °C. The indexed libraries were purified using a Gel/PCR DNA Fragments Extraction Kit (Geneaid Biotech, Xinpei, Taiwan) via column purification, and DNA concentrations were quantified using the Qubit^®^ dsDNA HS Assay Kit (Thermo Fisher Scientific, Waltham, USA). Final libraries were diluted to the required concentration and pooled for sequencing.

Raw sequencing data were subjected to quality control using FastQC v0.11.9 (49). Low-quality reads and adapter sequences were trimmed usingCutadapt v.3.4 and fastp (50, 51). Then, the reads were corrected using Bloocoo (52). High-quality reads were assembled into contigs using MEGAHIT v1.2.9 (automatic k-mer size selection) and SPAdes v.3.15.3 (error correction turned off) (53, 54). The assemblies were merged using GAM-NGS (55). Antimicrobial resistance (AMR) genes were predicted using ABRicate (CARD and NCBI databases) and the results were compared to RGI tool of the Comprehensive Antibiotic Resistance Database (CARD) (Seemann T, Abricate, Github https://github.com/tseemann/abricate).

To assess the potential mobility of the identified ARGs, we used MobileElementFinder v1.0.3 (56), which predicts mobile genetic elements (MGEs) present on the contigs. ARGs located within a species-specific distance from known MGEs—based on the longest composite transposon distance reported in the database—were considered potentially mobile. In addition, plasmid origin was predicted using PlasFlow v1.1 (57). For plasmid prediction, we applied the recommended threshold of 0.7, above which contigs were classified as plasmid derived.

For statistical evaluation, we filtered the results to include only those ARGs with sequence identity and coverage above 75%. The annotated resistance mechanism, a brief description, and associated antimicrobial classes were extracted from the CARD database to evaluate the clinical and epidemiological relevance of each gene.

## Results

### Phenotypic resistance profiles

The minimum inhibitory concentration (MIC) values obtained in this study are summarized in Tables 1, 2. According to EFSA-established microbiological cut-off values, the *Enterococcus faecium* strain exhibited resistance to amoxicillin (16 μg/mL), tylosin (4 μg/mL), and gentamicin (64 μg/mL), but remained susceptible to clindamycin. Based on CLSI clinical breakpoints, the strain was susceptible to penicillin, amoxicillin-clavulanic acid, and doxycycline, whereas resistance was detected against potentiated sulfonamides (8 μg/mL) and florfenicol (8 μg/mL).

**Table 1:** Minimum inhibitory concentration (MIC) values of the probiotic strains tested.

**No**.	**Strains**	**PEN**	**AMX**	**AMK**	**CTR**	**GEN**	**OTC**	**DOX**	**CLI**
			**MIC (**μ**g/mL)**						
1	*Enterococcus faecium*	8	16	4	32	64	4	1	2
2	*Bacillus licheniformis*	32	16	2	32	2	16	8	1
3	*Bacillus subtilis*	8	8	2	32	64	0.5	0.06	4
4	*Lactobacillus rhamnosus*	4	2	0.5	32	64	0.5	0.125	4
5	*Pediococcus acidilactici*	1	16	8	32	4	4	0.5	0.06

PEN, penicillin; AMX, amoxicillin; AMK, amoxicillin-clavulanic acid (2:1 ratio); CTR, ceftriaxone; GEN, gentamicin; OTC, oxytetracycline; DOX, doxycycline; CLI, clindamycin.

**Table 2:** Minimum inhibitory concentration (MIC) values of the probiotic strains tested (continued).

**No**.	**Strains**	**PSA**	**GAT**	**FLO**	**TIL**	**TIA**	**MET**	**SUL**	**TRM**
			**MIC (**μ**g/mL)**						
1	*Enterococcus faecium*	8	2	8	4	128	64	256	256
2	*Bacillus licheniformis*	0.5	0.5	8	0.25	128	64	128	1
3	*Bacillus subtilis*	8	0.5	8	4	128	64	256	128
4	*Lactobacillus rhamnosus*	8	1	8	4	128	64	64	64
5	*Pediococcus acidilactici*	8	0.5	4	0.125	1	64	64	256

PSA, potentiated sulfonamide (trimethoprim and sulfamethoxazole in 1:19 ratio); GAT, gatifloxacin; FLO, florfenicol; TIL, tilozin; TIA, tiamulin; MET, metronidazole; SUL, sulfamethoxazole; TRM, trimethoprim.

Among the *Bacillus* strains, *Bacillus licheniformis* was susceptible to both gentamicin and clindamycin, while *Bacillus subtilis* exhibited resistance to these antibiotics. *Lactobacillus rhamnosus* was susceptible to amoxicillin but resistant to gentamicin (64 μg/mL) and clindamycin (4 μg/mL). The *Pediococcus acidilactici* strain was susceptible to gentamicin and clindamycin, but resistant to amoxicillin (16 μg/mL).

Regarding the tetracycline class, low MIC values were observed for oxytetracycline in *Bacillus subtilis* (0.5 μg/mL) and *Lactobacillus rhamnosus* (0.5 μg/mL), and for doxycycline in *Bacillus subtilis* (0.06 μg/mL), *Lactobacillus rhamnosus* (0.125 μg/mL), and *Pediococcus acidilactici* (0.5 μg/mL). MIC values for gatifloxacin were as follows: *Enterococcus faecium* −2 μg/mL, *Bacillus licheniformis* −0.5 μg/mL, *Bacillus subtilis* −0.5 μg/mL, *Lactobacillus rhamnosus* −1 μg/mL, and *Pediococcus acidilactici* −0.5 μg/mL. The quality control strain's MIC values were within the CLSI-recommended QC ranges: penicillin 2 μg/mL, amoxicillin 0.5 μg/mL, amoxicillin–clavulanic acid 0.5 μg/mL, gentamicin 32 μg/mL, clindamycin 4 μg/mL, florfenicol 0.5 μg/mL, tylosin 2 μg/mL, and potentiated sulfonamide 0.5 μg/mL.

### Genotypic resistance profiles

**Figure 1:**
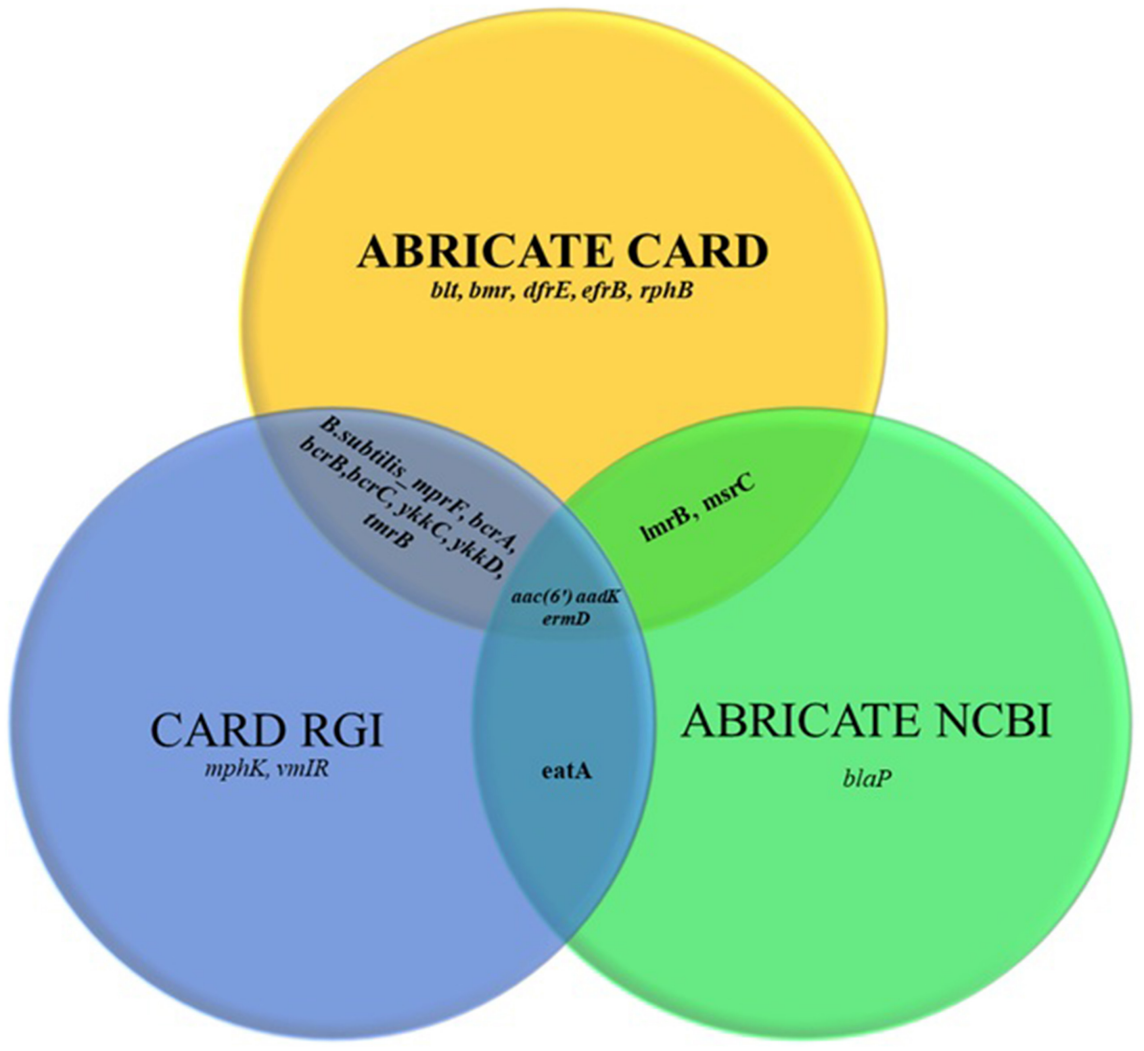
Comparison of the Comprehensive Antibiotic Resistance Database (CARD) ABRICATE, the CARD Resistance Gene Identifier (RGI) and the National Center for Biotechnology Information (NCBI) ABRICATE databases in terms of antimicrobial resistance gene sets.

The ARGs identified in the tested strains are summarized in Table 3. In total, 21 distinct ARG types were detected, with a cumulative 27 occurrences across the strains, indicating that certain ARGs were present in more than one isolate (Supplementary material). Comparison across three approaches (Figure 1) showed that the highest number of resistance genes (17) was detected using the ABRicate tool with the CARD database, followed by the CARD online Resistance Gene Identifier (RGI) interface (13 genes), while the ABRicate tool with the NCBI database yielded the fewest matches (7 genes).

**Table 3:** Antimicrobial resistance gene set identified during next-generation sequencing of probiotic strains.

**Strain**	**Coverage%**	**Identity %**	**ARG**	**Taxon**	**Plasmid**	**Phage**	**MGE**
*Enterococcus faecium*	100.00	98.36	*AAC(6′)-Ii*	*Enterococcus*	+	–	–
*Enterococcus faecium*	100.00	97.23	*msrC*	*Enterococcus*	–	–	–
*Enterococcus faecium*	95.76	75.43	*efrB*	*Enterococcus*	–	–	–
*Enterococcus faecium*	97.19	77.75	*dfrE*	*Enterococcus*	–	–	–
*Enterococcus faecium*	100.00	100.00	*eatA*	*Enterococcus*	–	–	–
*Bacillus licheniformis*	100.00	99.15	*bmr*	*Bacillus licheniformis*	–	–	–
*Bacillus licheniformis*	100.00	98.19	*lmrB*	*Bacillus licheniformis*	–	–	–
*Bacillus licheniformis*	100.00	99.83	*tmrB*	*Bacillus licheniformis*	–	–	–
*Bacillus licheniformis*	97.44	80.26	*rphB*	*Bacillus licheniformis*	–	–	–
*Bacillus licheniformis*	100.00	98.83	*mprF*	*Bacillus licheniformis*	–	–	–
*Bacillus licheniformis*	100.00	99.09	*blt*	*Bacillus licheniformis*	–	–	–
*Bacillus licheniformis*	99.88	98.83	*aadK*	*Bacillus licheniformis*	–	–	–
*Bacillus licheniformis*	100.00	100.00	*ykkC*	*Bacillus licheniformis*	–	–	–
*Bacillus licheniformis*	100.00	100.00	*blaP*	*Bacillus subtilis*	–	–	–
*Bacillus licheniformis*	100.00	99.69	*ykkD*	*Bacillus licheniformis*	–	–	–
*Bacillus licheniformis*	98.68	98.68	*mphK*	*Bacillus licheniformis*	–	–	–
*Bacillus licheniformis*	98.91	98.91	*vmr*	*Bacillus licheniformis*	–	–	–
*Bacillus subtilis*	97.21	79.51	*rphB*	*Bacillus subtilis*	–	–	–
*Bacillus subtilis*	100.00	99.31	*ermD*	*Bacillus subtilis*	–	–	–
*Bacillus subtilis*	100.00	97.23	*bcrC*	*Bacillus subtilis*	–	–	–
*Bacillus subtilis*	100.00	97.77	*bcrB*	*Bacillus subtilis*	–	–	–
*Bacillus subtilis*	100.00	98.59	*bcrA*	*Bacillus subtilis*	–	–	–
*Lactobacillus rhamnosus*	100.00	98.36	*AAC(6′)-Ii*	*Lactobacillus rhamnosus*	–	–	–
*Lactobacillus rhamnosus*	100.00	97.23	*msrC*	*Lactobacillus rhamnosus*	–	–	–
*Lactobacillus rhamnosus*	95.76	75.43	*efrB*	*Lactobacillus rhamnosus*	–	–	–
*Lactobacillus rhamnosus*	97.19	77.75	*dfrE*	*Lactobacillus rhamnosus*	–	–	–
*Lactobacillus rhamnosus*	100.00	100.00	*eatA*	*Lactobacillus rhamnosus*	–	–	–
*Pediococcus acidilactici*	–	–	–	–	–	–	–

ARG, Antibiotic resistance gene; MGE, Mobile genetic elements.

The mobility analysis indicated that only one ARG was predicted to be plasmid-associated, *aac(6*′*)-Ii* in *Enterococcus faecium* (Supplementary Table S3). This gene was located on a contig classified as plasmid-derived by PlasFlow (probability = 0.82), while MobileElementFinder did not identify any adjacent insertion sequences. A schematic representation of this contig is shown in Supplementary Figure S1.

**Figure 2:**
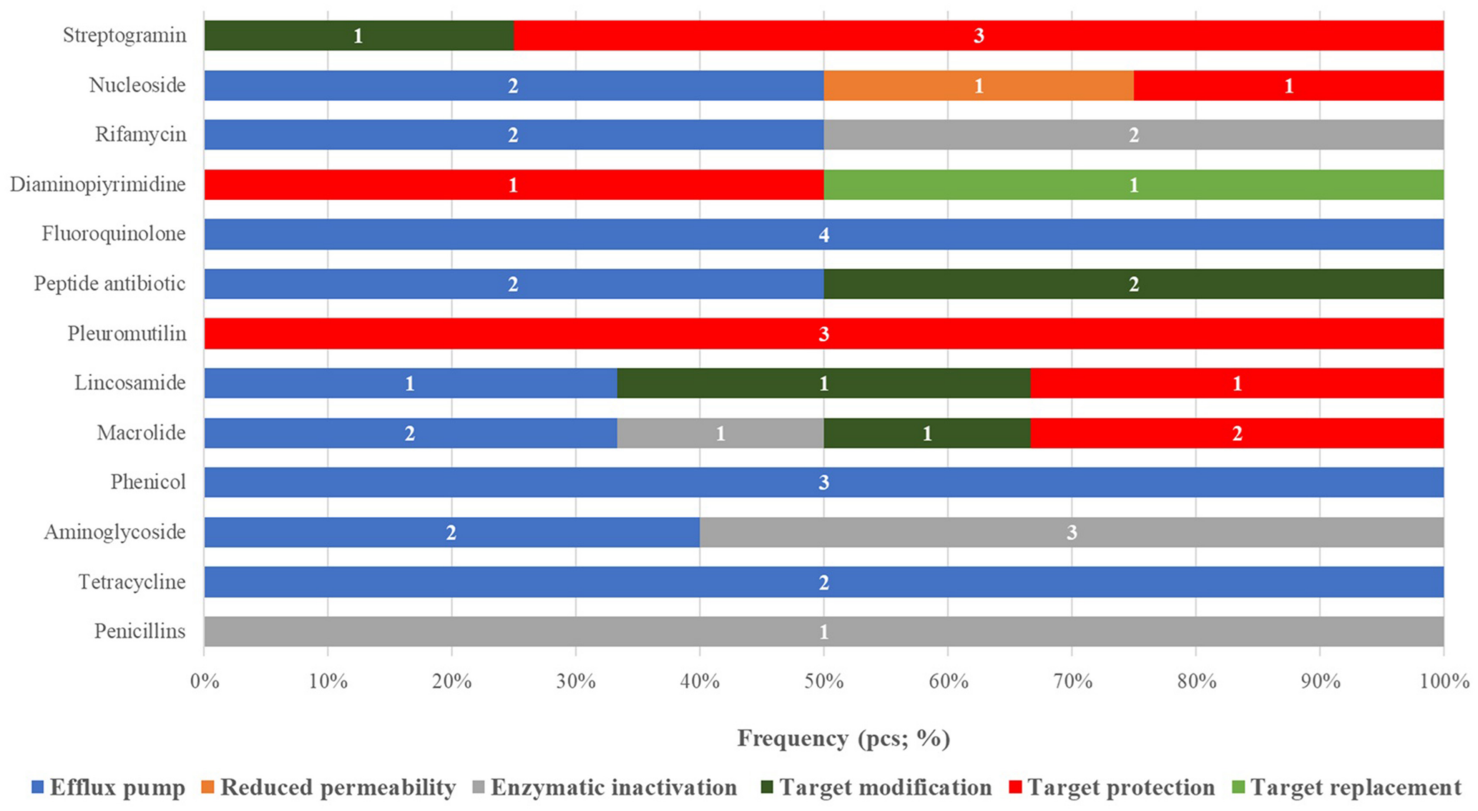
Classification of antimicrobial resistance genes identified in probiotic strains according to drug classes and resistance mechanisms, by frequency.

When categorizing the identified ARGs by resistance mechanism and antibiotic class (Figure 2), a total of 13 resistance pathways were recognized. Efflux pump mechanisms were the most prevalent (20 occurrences), followed by target protection mechanisms (11 occurrences). The highest number of ARGs was associated with macrolide resistance (6 instances).

On a strain-specific basis, *Bacillus licheniformis* harbored the broadest resistance gene profile (22 occurrences), predominantly related to efflux pump mechanisms. In contrast, *Pediococcus acidilactici* did not carry any detectable ARGs.

## Discussion

A comparison of the phenotypic resistance profiles and the identified ARGs revealed several cases of direct concordance, reinforcing the predictive value of genotypic data.

In the case of the *Enterococcus faecium* strain, the high MIC for gentamicin (64 μg/mL) could be well-explained by the presence of the *AAC(6*′*)-Ii* gene, an aminoglycoside acetyltransferase commonly associated with gentamicin resistance. Additionally, resistance to tylosin is supported by the detection of the *msrC* gene, encoding an ABC-type efflux pump linked to macrolide resistance. In a 2021 study by Sabna et al., *Enterococcus faecium* isolates were resistant to penicillin, ampicillin, erythromycin, kanamycin, and streptomycin, while remaining susceptible to tetracyclines, chloramphenicol, and rifampicin (58). Our findings similarly indicated amoxicillin resistance—an agent considered equivalent to ampicillin—and susceptibility to tetracyclines (i.e., doxycycline). A historical strain isolated circa 1920, still used as a probiotic in both humans and animals, was shown to remain susceptible to penicillin, erythromycin, tetracycline, chloramphenicol, and gentamicin, but demonstrated intrinsic resistance to oxacillin, kanamycin, and streptomycin. However, this strain remained susceptible to the penicillin–streptomycin combination (59). In our study, the tested strain was susceptible to penicillin but resistant to both amoxicillin and gentamicin. The observed difference between penicillin and amoxicillin susceptibility can be attributed to the use of epidemiological cut-off values (ECOFFs) by the EFSA panel, rather than clinical breakpoints. These ECOFFs are designed to distinguish between wild-type and non-wild-type populations and can indicate the emergence of reduced susceptibility within a population.

The detection of *AAC(6*′*)-Ii, msrC*, and *efrB* (multidrug resistance-associated) genes in the *Enterococcus faecium* isolate is particularly relevant from a probiotic safety perspective. These genes confer broad-spectrum resistance and, although the strains may be applied for probiotic use, the potential plasmid-borne nature of the *AAC(6*′*)-Ii* gene suggests a non-negligible risk of HGT to pathogenic gut bacteria. The presence of the *dfrE* (dihydrofolate reductase) and *eatA* genes further suggests that even isolates derived from healthy animals may harbor clinically significant resistance mechanisms. Although the majority of detected ARGs were chromosomally encoded, one plasmid-associated gene, *aac(6*′*)-Ii*, was identified in *Enterococcus faecium*. This finding highlights the potential for horizontal gene transfer, albeit limited in our dataset. The absence of phage-related or other MGEs suggests a relatively stable genetic background with low mobility potential for most of the resistance determinants.

In *Bacillus licheniformis*, the detected ARGs provide critical insight into potential antimicrobial hazards, even in strains intended for probiotic use. Of particular concern is the presence of *bmr* and *blt*, which are members of the major facilitator superfamily (MFS) efflux systems and confer resistance against a broad range of antimicrobials, including fluoroquinolones and chloramphenicol. The *aadK* gene, encoding an aminoglycoside nucleotidyltransferase, suggests resistance to key agents such as gentamicin, which was also reported by Agers et al. in 2019 (60). The detection of *ykkC* and *ykkD*, the two subunits of the ykkCD efflux system, further reinforces the risk of multidrug resistance. This pump system was also previously identified in *Bacillus subtilis* by Agers et al. (60).

In *Bacillus subtilis*, the identification of several ARGs also warrants attention. The *rphB* gene is linked to rifampin resistance via phosphotransferase activity. The *ermD* gene is responsible for the macrolide-lincosamide-streptogramin B (MLSB) resistance phenotype, a well-known resistance mechanism affecting macrolide efficacy. The *bcrC, bcrB*, and *bcrA* genes confer resistance to bacitracin; *bcrC* functions as an undecaprenyl pyrophosphate phosphatase when overexpressed, while *bcrB* and *bcrA* act as ABC transporters, enhancing resistance. This efflux system has been previously characterized in *Bacillus licheniformis* (60). Given the widespread use of *B. subtilis* in probiotic applications, resistance to bacitracin is of particular concern for food chain safety. Clindamycin resistance observed in this strain could be attributed to the *lmrB* efflux pump, previously described by Yoshida et al. in 2004 (61). Interestingly, while phenicol resistance (e.g., to florfenicol) was also observed, no specific florfenicol-resistance gene was identified. However, the *bmr* (62, 63) or *blt* MFS efflux systems (64) may partially account for the observed resistance, as they are known to contribute to multidrug resistance phenotypes. Although florfenicol and clindamycin resistance were observed in *Bacillus subtilis*, no canonical resistance genes (*floR/fex/cfr* or *erm/lnu*) were detected in our ARG-focused annotation. This discrepancy may be explained by intrinsic mechanisms such as chromosomally encoded multidrug efflux systems (e.g., *bmr/blt* transporters), which are widely distributed in *Bacillus* spp. We cannot exclude the possibility of additional mechanisms, including regulatory changes or target site mutations, which were not systematically assessed in this study. This represents a limitation of our analysis and an avenue for future research.

In *Lactobacillus rhamnosus*, genomic screening revealed multiple antimicrobial resistance genes (ARGs) that may represent a potential risk in terms of HGT. The *AAC(6*′*)-Ii* gene, typically associated with *Enterococcus* species, is a chromosomal aminoglycoside acetyltransferase that confers resistance to aminoglycosides. The *msrC* gene encodes an ABC-type efflux pump responsible for resistance to erythromycin, other macrolides, and streptogramin B antibiotics. The *efrB* gene, part of the *efrAB* efflux pump complex, plays a role in multidrug resistance. The *dfrE* gene encodes a dihydrofolate reductase that enables resistance to trimethoprim. Together, these genes highlight the potential hazards posed by probiotic *Lactobacillus rhamnosus* strains, particularly regarding the risk of HGT and the emergence of multidrug resistance.

The phenotypically observed resistance to gentamicin and clindamycin in *Lactobacillus rhamnosus* is consistent with genotypic findings: *AAC(6*′*)-Ii* may account for aminoglycoside resistance, while *msrC* likely mediates efflux-based resistance to macrolides and lincosamides. Resistance to oxacillin and cephalosporins has also been reported in *Lactobacillus plantarum* and *Lactobacillus rhamnosus* strains isolated from fermented foods (65, 66). Moreover, strains of both *Lactobacillus rhamnosus* and *Lactobacillus plantarum* have shown resistance to vancomycin, although the underlying mechanisms remain unclear (67, 68). Mater et al. demonstrated that *Lactobacillus acidophilus*, a widely used strain in the food industry, can acquire vancomycin resistance genes from *Enterococcus* species under both *in vitro* and *in vivo* conditions (69). While most *Lactobacillus* species remain susceptible to fluoroquinolones and phenicols, chloramphenicol resistance genes, some of which are mobile, have been identified in *Lactobacillus plantarum* from fermented vegetables, along with cross-resistance to erythromycin (70).

Among the most frequently reported ARGs in *Lactobacillus* species are those conferring resistance to tetracyclines and erythromycin. The *tetM* and *tetS* genes encode ribosomal protection proteins and may be chromosomally or plasmid located. The *tetL* gene encodes an efflux pump protein and is found exclusively on plasmids. Similarly, the erythromycin resistance gene *ermB* is plasmid-borne (71). Strains carrying multiple tetracycline resistance genes tend to exhibit stronger resistance phenotypes, likely due to synergistic interactions among these genes (72). Feld et al. identified an *Lactobacillus plantarum* strain harboring a transposon-borne *tetM* gene that was capable of transferring to other lactic acid bacteria (73).

In contrast, *Limosilactobacillus fermentum* is generally considered safe, as it lacks transferable ARGs and virulence genes (74). However, like many lactic acid bacteria, this species likely possesses intrinsic resistance to kanamycin and streptomycin (75). Lactic acid bacteria have been used for centuries in traditional food fermentation, where they are maintained through continuous subculturing, and are also employed as starter cultures in modern food biotechnology (76, 77). More recently, certain strains have been explored as live vaccine candidates or as starter cultures in animal feed formulations (78–80). Their broad application underscores not only their economic importance but also the need for responsible and cautious use, particularly with regard to ARG dissemination risks.

For *Bacillus licheniformis* and *Pediococcus acidilactici*, the low MIC values detected phenotypically, and the absence (or limited presence) of relevant resistance genes were in concordance, reinforcing the idea that genomic data can be predictive, not always correlate completely with phenotypic characteristics. Resistance gene expression, regulatory mechanisms, and environmental factors all influence phenotypic resistance outcomes. Du et al. reported that *Bacillus amyloliquefaciens* strains exhibited marked sensitivity to fluoroquinolones—especially enrofloxacin and ciprofloxacin—while older antimicrobial classes showed limited efficacy (37). In studies involving Tibetan yak isolates, most strains were fully susceptible to all antibiotics tested; only one strain was resistant to neomycin, and two were borderline for polymyxin B (81). In another study of *Bacillus licheniformis* from yaks, resistance to lincomycin was observed phenotypically, yet no corresponding resistance gene was detected via PCR, highlighting discrepancies between phenotype and genotype (82). This supports the notion that resistance genes may not always be detectable, potentially due to sequencing limitations or incomplete databases. Similarly, Dindhoria et al. found no ARGs in *Bacillus licheniformis* genomes using the CARD database (83).

For *Pediococcus acidilactici*, our strain was sensitive to gentamicin and clindamycin but showed resistance to amoxicillin. Zhao et al. reported complete susceptibility to piperacillin, imipenem, chloramphenicol, and erythromycin, while their strains showed moderate resistance to clindamycin, doxycycline, and levofloxacin, and complete resistance to vancomycin and tetracycline (38). Yang et al. tested the antimicrobial profiles of two probiotic strains used in mud crab studies. The *Pediococcus pentosaceus* strain showed resistance only to ceftazidime and sulfamethoxazole, while an *Enterococcus faecalis* strain was resistant to nearly all tested agents (84). These results suggest that *Pediococcus* strains may be safer alternatives for probiotic applications compared to *Enterococcus* species.

Across all strains, we identified 27 ARG occurrences representing 21 distinct resistance genes from the whole-genome data of the probiotic isolates. These findings confirm that even strains intended for veterinary probiotic use may harbor genes capable of contributing to antimicrobial resistance dissemination between the microbiome and the environment.

When comparing databases, the CARD ABRICATE tool proved to be the most accurate (17 genes), followed by the CARD online RGI (13 genes) and the NCBI ABRICATE (7 genes). This emphasizes the importance of database selection in genomic resistance studies, with CARD being clearly preferable for profiling ARGs in probiotics. Discrepancies in gene detection between databases were due to differences in database content. For example, sequences such as *blaP* fulfilled our thresholds for coverage and identity but were not detected in the NCBI AMR database because this resource is less frequently updated and contains a narrower gene set compared with CARD or ResFinder. This highlights the importance of using multiple databases for ARG annotation to minimize under-detection bias.

Mechanistically, efflux pump-related genes were the most dominant (20 occurrences), underlining their central role in multidrug resistance (MDR) among probiotic strains. This was followed by target protection mechanisms (11 occurrences), which modify the antibiotic's molecular binding site and are especially relevant in macrolide resistance (6 occurrences).

Among species, *Bacillus licheniformis* harbored the most ARGs (22 total), predominantly efflux-related, highlighting the strain's potentially large intrinsic resistome and its possible role as a gene donor via HGT. In contrast, *Pediococcus acidilactici* had no detectable ARGs, suggesting a naturally sensitive profile and a safer probiotic candidate from a resistance standpoint.

This study demonstrates that the resistance profiles of probiotic strains are shaped by complex genetic backgrounds and multifactorial influences. For veterinary applications, ARG mobilization potential and HGT risk must be evaluated thoroughly. Our findings stress that probiotic strain selection and utilization must consider not only beneficial traits but also their genomic resistance repertoire. These insights are essential for future regulatory and authorization frameworks, especially in the context of the One Health approach and global efforts to control antimicrobial resistance.

## Conclusion

This study provides a comprehensive overview of the antimicrobial resistance profiles, both phenotypic and genotypic of commonly used probiotic bacterial strains (*Enterococcus faecium, Bacillus licheniformis, Bacillus subtilis, Lactobacillus rhamnosus*, and *Pediococcus acidilactici*). MIC testing revealed resistance to several clinically important antibiotics in multiple strains, including amoxicillin, gentamicin, clindamycin, tylosin, and florfenicol. Whole-genome sequencing identified 27 distinct resistance genes associated with diverse mechanisms, such as efflux pumps, target modification, target protection, and antibiotic-inactivating enzymes.

Of particular concern was *Bacillus licheniformis*, which harbored the broadest spectrum of resistance genes, including multiple efflux systems associated with multidrug resistance. In contrast, *Pediococcus acidilactici* did not possess any identifiable ARGs. In several cases, phenotypic susceptibility aligned with genotypic predictions, although discrepancies were observed, likely reflecting cryptic or regulated resistance mechanisms. Among the database–tool combinations tested, ABRicate run with the CARD database yielded the most reliable ARG identification, whereas ABRicate with the NCBI database returned the fewest matches, highlighting the critical importance of database selection in genomic AMR profiling.

Our findings underscore that probiotic strains, while beneficial, may also serve as potential reservoirs of antimicrobial resistance genes, especially when introduced into the food chain. This highlights the need for comprehensive AMR screening, even for strains classified as generally regarded as safe (GRAS). Integrating *in vitro* susceptibility testing with genomic-based prediction represents an effective strategy for risk assessment and ensuring the safe use of probiotics in veterinary and food applications.

## Data Availability

The datasets analyzed for this study can be found in the National Library of Medicine: https://www.ncbi.nlm.nih.gov/bioproject/PRJNA1303120.
