# Supplementary material for: Phenotypic and genotypic characterization of probiotic strains in the context of antimicrobial resistance
Source: Front Vet Sci. 2025 Oct 3;12:1684650. doi: 10.3389/fvets.2025.1684650 (PMC12533545; doi:10.3389/fvets.2025.1684650)
Supplement: Supplementary file 2 [file Table_2.docx]

Supplementary Material

**Supplementary Table 1.** Summary of minimum inhibitory concentrations (MIC) breakpoints (μg/mL) applied for resistance/susceptibility interpretation.

| **Bacteria** | **Microbiological cut-off values of European Food Safety Authority (µg/ml)** | | | | | **CLSI (µg/ml)** |
| --- | --- | --- | --- | --- | --- | --- |
|  | Amoxicillin | Gentamicin | Clindamycin | Tylosin | Penicillin | |
| *Enterococcus faecium* | 2 | 32 | 4 | 4 | 16 | |
| *Bacillus licheniformis* | n.a. | 4 | 4 | n.a. | n.a. | |
| *Bacillus subtilis* | n.a. | 4 | 4 | n.a. | n.a. | |
| *Lactobacillus rhamnosus* | 4 | 16 | 4 | n.a. | n.a. | |
| *Pediococcus acidilactici* | 4 | 16 | 1 | n.a. | n.a. | |

CLSI – Clinical and Laboratory Standards Institute; n.a. – not available

**Supplementary Table 2.** Sequencing library preparation parameters of probiotic bacterial isolates.

| **Sample** | **Bacteria** | **Index 1** | **Index 2** | **All read** |
| --- | --- | --- | --- | --- |
| B25 | *Enterococcus faecium* | N706 - TAGGCATG | S503 - TATCCTCT | 4,216,250 |
| B26 | *Bacillus licheniformis* | N707 - CTCTCTAC | S503 - TATCCTCT | 4,126,168 |
| B27 | *Bacillus subtilis* | N710 - CGAGGCTG | S503 - TATCCTCT | 3,673,914 |
| B31 | *Lactobacillus rhamnosus* | N711 - AAGAGGCA | S503 - TATCCTCT | 3,613,806 |
| B34 | *Pediococcus acidilactici* | N712 - GTAGAGGA | S503 - TATCCTCT | 3,717,668 |

**Supplementary Table 3.** Detailed results of mobile genetic element (MGE) analysis using MobileElementFinder and PlasFlow.

| **Strain** | **Sequence** | **ARG** | **MobileElementFinder** | **PlasFlow** | **PlasFlow probability** | **Mobility interpretation** |
| --- | --- | --- | --- | --- | --- | --- |
| *Enterococcus faecium* | PairedContig_120 | *aac(6’)-Ii* | none detected | plasmid | 0.82 | Plasmid-associated ARG |

**
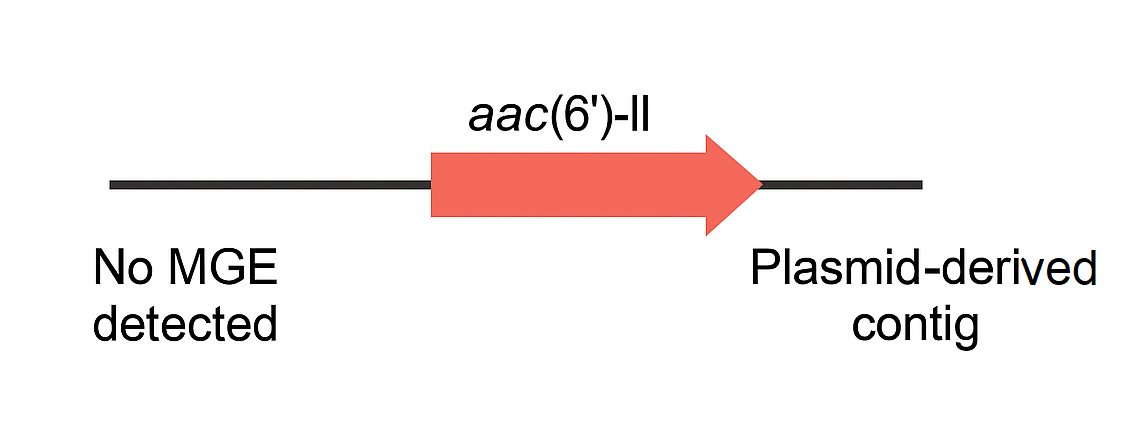
**

**Supplementary Figure 1.** Gene map showing the *aac(6’)-Ii* resistance gene found on a plasmid-derived contig in Enterococcus faecium strain. No mobile genetic elements (MGEs) were detected in its flanking regions.
